# Supplementary material for: Cerebellar and subcortical contributions to working memory manipulation
Source: Commun Biol. 2025 Jul 9;8:1028. doi: 10.1038/s42003-025-08467-0 (PMC12241541; doi:10.1038/s42003-025-08467-0)
Supplement: Supplementary file 3 — Description of Additional Supplementary Files [file 42003_2025_8467_MOESM3_ESM.pdf]

Description of Additional Supplementary Files

File name: Supplementary data 1
Description: Atlas labels for brain regions comparing Easy-Correct vs. Hard-Correct

File name: Supplementary data 2
Description: Atlas labels for brain regions comparing Hard-Correct vs. Hard-Incorrect

File name: Supplementary data 3
Description: Atlas labels for brain regions important for both comparisons
