# Supplementary material for: Cerebellar and subcortical contributions to working memory manipulation
Source: Commun Biol. 2025 Jul 9;8:1028. doi: 10.1038/s42003-025-08467-0 (PMC12241541; doi:10.1038/s42003-025-08467-0)
Supplement: Supplementary file 7 — Reporting Summary [file 42003_2025_8467_MOESM7_ESM.pdf]

Corresponding author(s): James M. Shine

Last updated by author(s): Mar 26, 2025

Reporting Summary

Nature Portfolio wishes to improve the reproducibility of the work that we publish. This form provides structure for consistency and transparency in reporting. For further information on Nature Portfolio policies, see our Editorial Policies and the Editorial Policy Checklist.

Statistics

For all statistical analyses, confirm that the following items are present in the figure legend, table legend, main text, or Methods section.

n/a	Confirmed	
☐	☒	The exact sample size (n) for each experimental group/condition, given as a discrete number and unit of measurement
☐	☒	A statement on whether measurements were taken from distinct samples or whether the same sample was measured repeatedly
☐	☒	The statistical test(s) used AND whether they are one- or two-sided<br>*Only common tests should be described solely by name; describe more complex techniques in the Methods section.*
☐	☒	A description of all covariates tested
☐	☒	A description of any assumptions or corrections, such as tests of normality and adjustment for multiple comparisons
☐	☒	A full description of the statistical parameters including central tendency (e.g. means) or other basic estimates (e.g. regression coefficient) AND variation (e.g. standard deviation) or associated estimates of uncertainty (e.g. confidence intervals)
☐	☒	For null hypothesis testing, the test statistic (e.g. F, t, r) with confidence intervals, effect sizes, degrees of freedom and P value noted<br>*Give P values as exact values whenever suitable.*
☒	☐	For Bayesian analysis, information on the choice of priors and Markov chain Monte Carlo settings
☒	☐	For hierarchical and complex designs, identification of the appropriate level for tests and full reporting of outcomes
☐	☒	Estimates of effect sizes (e.g. Cohen's d, Pearson's r), indicating how they were calculated

Our web collection on statistics for biologists contains articles on many of the points above.

Software and code

Policy information about availability of computer code

Data collection	Data was originally collected by King and colleagues (https://www.nature.com/articles/s41593-019-0436-x). The 26 task were presented and behavioral data collected using custom-written code in MATLAB R2015b. The low-level functionality from psychtoolbox (3.0.13) was used for display and execution of the tasks tested in both the behavioural and fMRI sessions.
Data analysis	Data was preprocessed using fMRIprep and Python package Nilearn. Analysis of both the behavioral and functional MRI data was conducted in MATLAB v2022b. Code required to reproduce the statistical analyses and figures are publicly available at https://github.com/ShineLabUSYD/WM_Manipulation

For manuscripts utilizing custom algorithms or software that are central to the research but not yet described in published literature, software must be made available to editors and reviewers. We strongly encourage code deposition in a community repository (e.g. GitHub). See the Nature Portfolio guidelines for submitting code & software for further information.

Data

Policy information about availability of data

All manuscripts must include a data availability statement. This statement should provide the following information, where applicable:

- Accession codes, unique identifiers, or web links for publicly available datasets
- A description of any restrictions on data availability
- For clinical datasets or third party data, please ensure that the statement adheres to our policy

All imaging and behavioral data are publicly available in an OpenNeuro repository (accession number ds002105). The experimental and analysis code is available on a code-sharing website (https://github.com/maedbhk/MDTB-Cerebellum).

Research involving human participants, their data, or biological material

Policy information about studies with human participants or human data. See also policy information about sex, gender (identity/presentation), and sexual orientation and race, ethnicity and racism.

Reporting on sex and gender	In the original study, 24 young adults (16 women, 8 men; mean age = 23.8 years, sd = 2.6) were analysed (2 participants excluded due to failure to complete all scans). The research question of this study was related to how the brain can operationalise working memory manipulation which we believe to our knowledge is not affected by sex or gender, hence no analyses directly comparing either sex or gender was implemented.
Reporting on race, ethnicity, or other socially relevant groupings	The study was conducted in Western University, Canada. Undergraduate and graduate students were recruited and all participants were treated as a single group. Otherwise, there were no specific race, ethnic, or social groupings.
Population characteristics	The final sample consisted of 24 healthy, right-handed individuals (16 females, 8 males; mean age=23.8 years old, SD=2.6) with no self-reported history of neurological or psychiatric illness.
Recruitment	Undergraduate and graduate students were recruited (via posters) from the larger student body at Western University. Thus, our sample was biased towards relatively high-functioning, healthy and young individuals. While we don't expect cerebellar organization to be dramatically different in this group, caution needs to be exercised when generalizing the results to the general population.
Ethics oversight	The Ethics committee at Western University approved all experimental protocols (Protocol number: 107293)

Note that full information on the approval of the study protocol must also be provided in the manuscript.

Field-specific reporting

Please select the one below that is the best fit for your research. If you are not sure, read the appropriate sections before making your selection.

☒ Life sciences ☐ Behavioural & social sciences ☐ Ecological, evolutionary & environmental sciences

For a reference copy of the document with all sections, see nature.com/documents/nr-reporting-summary-flat.pdf

Life sciences study design

All studies must disclose on these points even when the disclosure is negative.

Sample size	No formal power analysis of the sample size was conducted. The sample size was taken from the original paper of King and colleagues (https://www.nature.com/articles/s41593-019-0436-x).
Data exclusions	For the scans of interest, 26 participants were originally recruited however 2 participants were excluded as they failed to complete all scanning runs.
Replication	Replication of the results were conducted at multiple levels: for classification, k-fold cross-validation was used to validate subsamples of the data produce consistent results. The results were also replicated across parcellations and spatial scales using higher spatial resolution parcellations.
Randomization	The sequence of task was randomized across imaging runs. All of the participants performed the same sequence of tasks (and the same number / order of runs) to enable analysis on the timeseries across participants.
Blinding	There was only the one group (no control or experimental group comparisons), therefore blinding was not applicable.

Reporting for specific materials, systems and methods

We require information from authors about some types of materials, experimental systems and methods used in many studies. Here, indicate whether each material, system or method listed is relevant to your study. If you are not sure if a list item applies to your research, read the appropriate section before selecting a response.

Materials & experimental systems

n/a	Involved in the study
☒	☐ Antibodies
☒	☐ Eukaryotic cell lines
☒	☐ Palaeontology and archaeology
☒	☐ Animals and other organisms
☒	☐ Clinical data
☒	☐ Dual use research of concern
☒	☐ Plants

Methods

n/a	Involved in the study
☒	☐ ChIP-seq
☒	☐ Flow cytometry
☐	☒ MRI-based neuroimaging

Plants

Seed stocks

Report on the source of all seed stocks or other plant material used. If applicable, state the seed stock centre and catalogue number. If plant specimens were collected from the field, describe the collection location, date and sampling procedures.

Novel plant genotypes

Describe the methods by which all novel plant genotypes were produced. This includes those generated by transgenic approaches, gene editing, chemical/radiation-based mutagenesis and hybridization. For transgenic lines, describe the transformation method, the number of independent lines analyzed and the generation upon which experiments were performed. For gene-edited lines, describe the editor used, the endogenous sequence targeted for editing, the targeting guide RNA sequence (if applicable) and how the editor was applied.

Authentication

Describe any authentication procedures for each seed stock used or novel genotype generated. Describe any experiments used to assess the effect of a mutation and, where applicable, how potential secondary effects (e.g. second site T-DNA insertions, mosiacism, off-target gene editing) were examined.

Magnetic resonance imaging

Experimental design

Design type

Task-based

Design specifications

Two task sets. 2 fMRI scanning sessions per task set. 8 functional imaging runs per session (10-min each). 17 tasks per imaging run (35 s each).

Behavioral performance measures

Variables recorded: response made, number of correct responses, false alarms, missed responses, response time. Accuracy (% correct) and reaction time (ms) were collected and averaged across tasks per participant.

Acquisition

Imaging type(s)

EPI, MPRAGE, and GRE field maps

Field strength

3T

Sequence & imaging parameters

EPI: Gradient echo, multi-band (factor 3, interleaved) with an in-plane acceleration (factor 2). Imaging parameters were: TR=1 sec, FOV=20.8cm, phase encoding direction was P to A, acquiring 48 slices with in-plane resolution of 2.5 mm x 2.5 mm and 3 mm thickness. For anatomical localization and normalization, a 5-min high-resolution scan of the whole brain was acquired (MPRAGE, FOV=15.6 cm x 24 cm x 24 cm, at 1x1x1 mm voxel size).

Area of acquisition

whole-brain

Diffusion MRI ☐ Used ☒ Not used

Preprocessing

Preprocessing software

Data preprocessing was conducted using fMRIprep which was set up on the University of Sydney High Performance Computer (HPC). Preprocessing was also conducted using a Python package Nilearn version 0.10.2. Processing of behavioural data was conducted in MATLAB v2022b

Normalization

All imaging data was co-registered to a standard template (MNI152nLin6Asym 2mm resolution). Then time series of parcellations were extracted from this space using the Schaefer atlas (cerebral cortical; https://pubmed.ncbi.nlm.nih.gov/28981612/), Tian atlas (subcortical; https://www.nature.com/articles/s41593-020-00711-6), SUIT atlas (cerebellum; https://pubmed.ncbi.nlm.nih.gov/16904911/).

Normalization template

Time series of parcellations were extracted from this space using the Schaefer atlas (cerebral cortical; https://

Normalization template

pubmed.ncbi.nlm.nih.gov/28981612/), Tian atlas (subcortical; https://www.nature.com/articles/s41593-020-00711-6), SUIT atlas (cerebellum; https://pubmed.ncbi.nlm.nih.gov/16904911/).

Noise and artifact removal

Noise and motion artifacts were removed by regressing out 12 head motion parameters (trans-, rot-x,y,z, including first derivatives). Confounding physiological signals regressed out include the combined cerebrospinal fluid (CSF) and white matter (WM)

Volume censoring

We excluded the first 3 volumes of each imaging run. Otherwise no volumes were censored.

Statistical modeling & inference

Model type and settings

We used generalised linear mixed effects models (GLMM; univariate) to estimate task related BOLD activity across time. We used linear distriminant analysis (multivariate) to classify spatial patterns between the conditions. We used cross-correlation (univariate) and attractor landscape analysis (univariate) to investigate temporal dynamics of the BOLD activity. For statistical analyses of behaviour and BOLD activity, generalised linear mixed effect models (univariate) were used.

Effect(s) tested

Effects tested included: trial onset, task difficulty (easy, medium, hard), task response (correct, incorrect)

Specify type of analysis: ☐ Whole brain ☐ ROI-based ☒ Both

Anatomical location(s)

Labels were predetermined based off the atlas. For cerebral cortical regions we used the Schaefer atlas (https://pubmed.ncbi.nlm.nih.gov/28981612/), for subcortical regions the Tian atlas (subcortical; https://www.nature.com/articles/s41593-020-00711-6), and for cerebellar regions the SUIT atlas (cerebellum; https://pubmed.ncbi.nlm.nih.gov/16904911/).

Statistic type for inference

(See Eklund et al. 2016)

Analyses were conducted at the whole-brain level, before narrowing down onto individual regions (parcellations) that were distributed amongst the whole brain

Correction

False Discovery Rate (FDR) correction was used to correct for using multiple univariate measurements to compare task with regions

Models & analysis

n/a	Involved in the study
☒	☐ Functional and/or effective connectivity
☒	☐ Graph analysis
☐	☒ Multivariate modeling or predictive analysis

Multivariate modeling and predictive analysis

GLMM: trial onset (window of time), task difficulty (easy, medium, hard), session (1-8).
LDA Classifier: Balanced Accuracy (average of specificity and sensitivity), combined with 5-fold cross-validation, iterating across including varying number of principal components and at different time points
